# Light-Induced In Situ Transmission Electron Microscopy—Development, Challenges, and Perspectives

**DOI:** 10.1021/acs.nanolett.2c03669

**Published:** 2022-11-28

**Authors:** Andrzej M. Żak

**Affiliations:** Wroclaw University of Science and Technology, Wybrzeże Wyspiańskiego 27, 50-370Wrocław, Poland

**Keywords:** optically coupled transmission electron microscopy, OTEM, in situ, TEM, photocatalysis, photodynamic, photoinduced

## Abstract

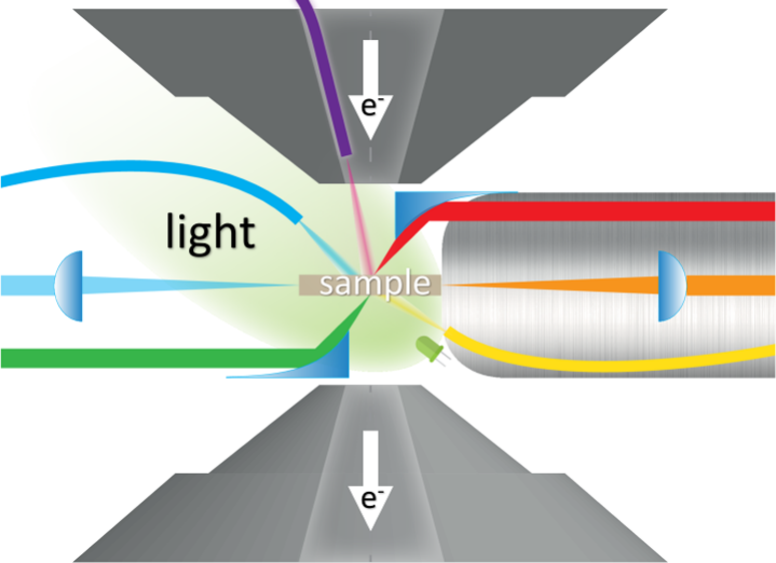

Transmission electron microscopy is a basic technique used for examining matter at the highest magnification scale available. One of its most challenging branches is in situ microscopy, in which dynamic processes are observed in real time. Among the various stimuli, like strain, temperature, and magnetic or electric fields, the light–matter interaction is rarely observed. However, in recent years, a significant increase in the interest in this technique has been observed. Therefore, I present a summary and critical review of all the in situ experiments performed with light, various technical possibilities for bringing radiation inside the transmission electron microscope, and the most important differences between the effects of light and electrons on the studied matter. Finally, I summarize the most promising directions for further research using light excitation.

Transmission electron microscopy (TEM) has had an unprecedented impact on the development of science since the first half of the 20th century. For many decades, we have had the opportunity not only to study the phenomena of interest at an atomic scale but also to influence matter during the observation. These types of experiments are called dynamic microscopy or, more commonly, in situ transmission electron microscopy.

In situ TEM is almost as old as the basic imaging technique itself. After all, the electron beam interacts with the matter intensively, changing its structure and properties with the varying dose. This phenomenon was first observed in 1934 by Laszlo Marton, who described damage to the biological cell under electron irradiation.^1^ In the next decades, a few solutions appeared that allowed the specimen to be influenced by, among others, temperature changes, electric and magnetic fields, the flux of accelerated particles, and liquids and gases in environmental TEM (ETEM). Advanced experiments often require the combination of several stimuli. Interested readers are referred to reviews generally dedicated to in situ TEM,^2^ nanoscale mechanical testing,^3^ ETEM,^4^ electrochemical liquid cells,^5^ magnetism-driven research,^6^ electrical interactions,^7^ and the influence of accelerated particles.^8^

Compared to other in situ TEM techniques, the interaction of light and matter lies outside of most researchers’ interests. There is little overlap between researchers involved in fluorescence or multi-photon microscopy and those specializing in the use of electron beams. This is because it is difficult to relate light-induced effects to real structural changes observable at the micro or nano level. The effects related to light absorption are usually described with the help of the Jabłoński diagram.^9^ A molecule excited by a quantum of light goes from the ground state to one of the vibrational levels of the excited singlet states. It can return to its original state by emission of a fluorescent photon or nonradiative relaxation, or undergo intersystem crossing to reach the excited triplet state. From this point, it can return to equilibrium by emission of a phosphorescent photon, by nonradiative relaxation, or by transferring its energy to another molecule. The last effect drives most of the electron-observable effects, like chemical reactions or the generation of gas bubbles from a liquid. Photochemistry offers a wealth of phenomena and effects to which electron imaging remains mostly insensitive. However, incomparably better TEM resolution can be achieved with appropriate experimental design.

Below, I would like to present a brief historical overview of the previous experiments and the various methods by which light is supplied in the high-vacuum area of the TEM sample. Studies using cathodoluminescent methods were intentionally omitted—in their case, light is generated in the sample as a result of the interaction with an electron beam and analyzed outside the microscope. This type of research has been extensively addressed in another review article.^10^ A topic related to light-induced research is the idea of ultrafast TEM, in which the sample could be illuminated, with the main advantage of the method being the generation of ultrafast electron beam pulses by photoemission from the electron cathode. This topic has also already been the addressed in several valuable reviews.^11,12^

Light was, for the first time, unintentionally applied to a TEM sample with the usage of heating stages during other in situ experiments. Above several hundred degrees Celsius, light is emitted from the holder and the sample itself via thermal emission, but its influence has always remained negligible. The first intentional combination of an electron microscope and a sample light illuminator was described in 1984 and concerned the effect of light on the movement of dislocations in CdS, CdSe, ZnO, and CdTe.^13^ It was also a rare approach in that, in addition to the optical fiber, a light bulb source was installed inside the column. The work indicated that illumination did not affect the structure of the studied materials, but the authors noticed that electron imaging could induce simultaneous processes similar to a light beam. Afterward, light-induced TEM studies had to wait over a decade for another chance. Then, in 1995, a combination of TEM and spectrofluorimetry was constructed,^14^ and the setup was tested on a diamond sample. Only 1% of the light emitted by the laser reached the sample, but the main task of the system was not to induce microstructural effects but to measure the photoluminescence in a selected area of the TEM sample, with simultaneous viewing using a light microscope. A similar concept, but fully integrated with the specimen holder, achieving sub-nanosecond temporal resolution, was also developed quite recently.^15^

A decade later, in 2004, researchers at Nagoya University used a proprietary system that led the optical fiber and 360 nm ultraviolet (UV) radiation near the sample and showed photodecomposition of polyhydrocarbons on catalytic TiO_2_ films.^16^ The authors noticed the increased visibility of the lattice fringes after 3 h of exposure, which was the result of reducing the number of amorphous hydrocarbons on the sample’s surface. This effect was confirmed by electron energy loss analysis (Figure 1a). This experiment is considered to be the first in situ observation of a light-induced process. Three years later, the same group extended the research with the use of ionic liquids, which allowed for diffusion testing under photocatalysis conditions and photonucleation of Au nanoparticles on TiO_2_.^17^

**Figure 1:**
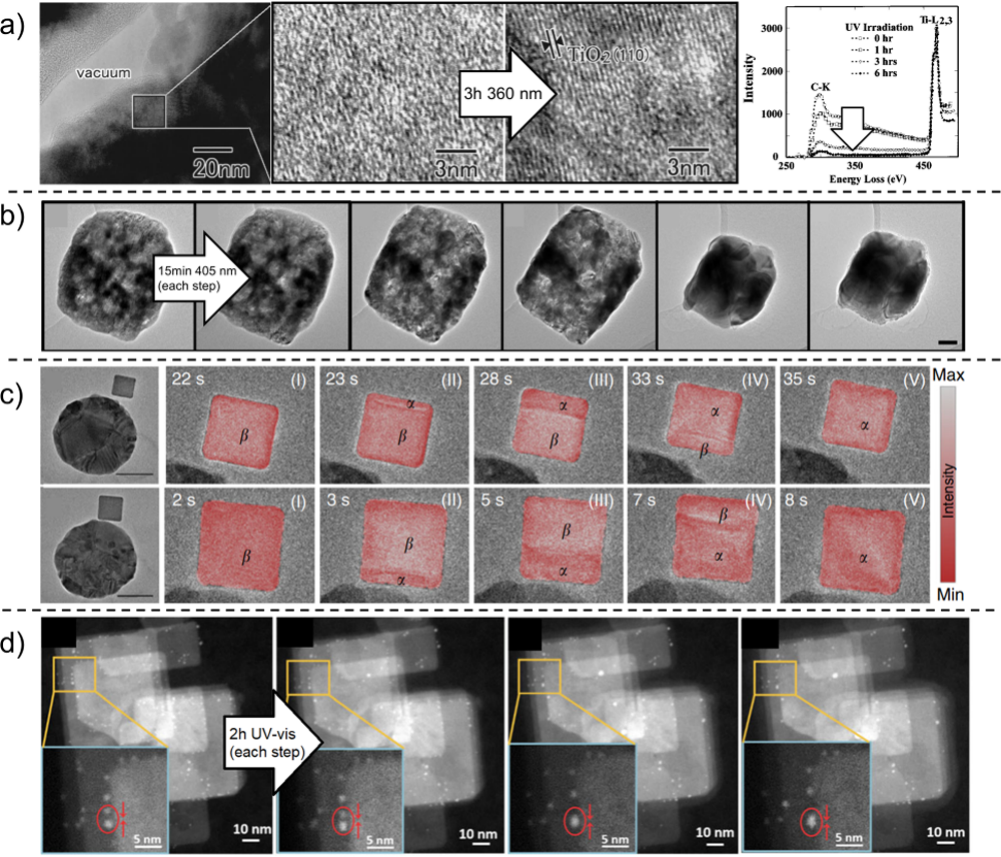
(a) Photodecomposition of polyhydrocarbons on TiO_2_, proved with high-resolution imaging and energy loss spectra. Adapted from ref (16) with permission. Copyright 2004 AIP Publishing. (b) Light-induced reduction of CuO into Cu in water vapor ETEM. Adapted from ref (21) with permission. Copyright 2013 Wiley-VCH Verlag GmbH& Co. (c) Pd-H β-to-α phase transformation under 690 nm light illumination in H_2_ ETEM. Adapted from ref (22) under CC-BY-4.0. Copyright 2018 Springer Nature. (d) Au clusters coalescence under Xe-lamp illumination. Adapted from ref (23) with permission. Copyright 2021 Springer Nature.

Research on photocurrents and mechanical interactions is a separate and highly specialized branch of light-induced experiments. They usually require the development of dedicated systems with a micromanipulator; this concept was used for the first time in 2009, when Shindo et al. published a setup of a specimen holder in which laser radiation was added to the double-probe piezodriving holder (Figure 2a) to investigate the discharging effect in commercial organic photoconductors.^18^ In the same year, Gao et al. used a light-emitting diode (LED) mounted in a specimen holder to show a direct relation between the bending of a ZnO nanowire and its photocurrent.^19^ The latter work combined mechanical action and light in one experiment for the first time. In another article published in the same year, Ohno et al. observed photoinduced movement of dislocations in ZnO^20^ using the system described previously.^14^

**Figure 2:**
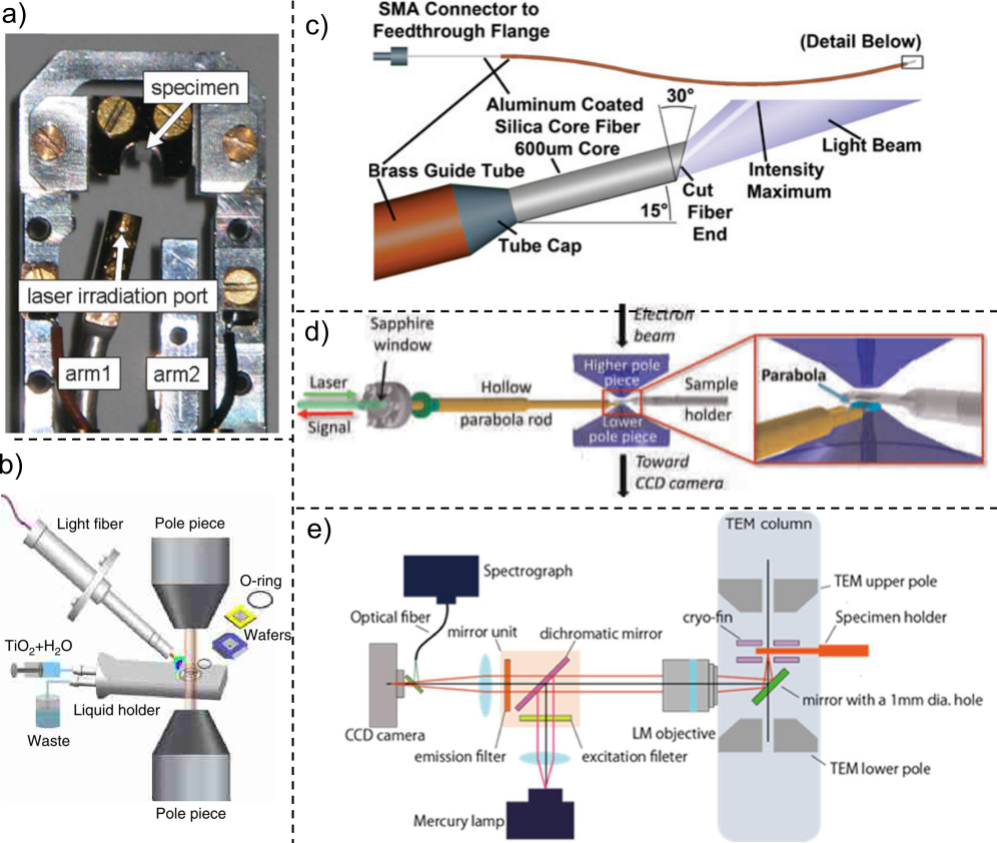
Different approaches to bring light into TEM. (a) Light fiber illuminator inside a TEM holder. Adapted from ref (18) with permission. Copyright 2009 Oxford University Press. (b) Fluidic TEM holder with UV illumination. Adapted from ref (37) under CC-BY-4.0. Copyright 2018 Springer Nature. (c) Light fiber mounted below the specimen inside a TEM pole piece. Adapted from ref (29) with permission. Copyright 2013 Cambridge University Press. (d) A parabolic mirror placed below the sample holder. Adapted from ref (34) with permission. Copyright 2015 Elsevier. (e) Light microscope used in TEM, with a mirror placed below the sample. Adapted from ref (41) with permission. Copyright 2021 Elsevier.

The real dawn of light-induced experiments took place after 2012. Two groups independently proposed the introduction of near-field optical probing,^24,25^ of which the undeniable advantage is the possibility of illuminating only the micro- or nanometric areas of the specimen. In one case, it was used to influence the dislocation structure of ZnO,^24^ and in the other, to crystallize amorphous silicon under the influence of high temperature caused by laser light.^25^ Even though in this case no typical photochemical effect other than the thermal effect was analyzed, the narrow-field illumination concept is very promising for further development. Another idea for introducing light through the specimen holder was reported by Cavalca et al.,^21^ who used a customized holder to observe the reduction of CuO to Cu under 405 nm radiation illumination and a water vapor environment in ETEM (Figure 1b). Another example presented by the same authors was the photodeposition of Pt on GaN:ZnO nanoparticles under similar ETEM conditions.^26^ A different, interesting solution appeared at Arizona State University,^27^ where a fragment of the heating holder’s frame was machined out, making it possible to lead the fiber from the side of the sample (Figure 2c).^28−30^ This approach was used to observe the photocatalytic activity of TiO_2_. Zhang et al. proposed the specimen holder setup with a piezoelectric-driven needle for simultaneous electrical measurements and illumination, which allowed the characterization of the optoelectronic properties of CdS/Si^31^ and CdS/ZnO heterostructures^32^ as well as in situ bending of an individual ZnO nanowire.^33^ Most of the above-mentioned works used dedicated sample holders, which were difficult to integrate with, e.g., heating or cooling devices. A solution to this problem was proposed by Picher et al.,^34^ who used a parabolic mirror below the sample, making the illuminator independent of the holder (Figure 2d), and showed that the system is capable of cathodoluminescence and Raman spectroscopy applications. The latter method was also the target of the tapered-fiber illuminator, which worked with an appropriately cut specimen holder that Allen et al. used for laser ablation and in situ Raman spectroscopy on a MoS_2_ flake.^35^ The trend toward narrowing the illumination field, noticeable in a large part of the aforementioned studies, is also important in another work,^36^ in which the laser illuminator was installed in a place typical for the energy-dispersive X-ray spectrometry (EDS) detector at a specified angle. In this case, the authors focused only on the thermal effect; however, this does not exclude the use of the device for observations of more sophisticated light-induced phenomena. A similar lighting system was used by Lu et al., who illuminated TiO_2_ in an aqueous liquid cell holder (Figure 2b) and observed the generation of H_2_ and the formation of a hydrogenated TiO_2_ shell, also around particles decorated with Pt.^37^ The authors used UV lighting with a fairly low intensity of 0.1 W/cm^2^, but their exposure times were up to 24 h. The idea of using an internal light source returned in 2019, when a modified specimen holder with a LED served as the basis for photoelectric measurements of TiO_2_ decorated with CdSe quantum dots (QDs) at the nanoscale.^38^

A practical breakthrough in light-induced research was the observation of phenomena related to localized surface plasmon resonance (LSPR). A group at Stanford University used aberration-corrected ETEM together with a cathodoluminescence specimen holder to observe the plasmon-dependent phase transformation of palladium nanoparticles.^22^ At relatively low mean light intensities of 1.5 to 3.8 mW/cm^2^, an acceleration of the phase transformation by an order of magnitude was observed when the particle was oriented within the range of the Au antenna. The experiment was conducted at controlled H_2_ pressure and temperature, which allowed the Pd nanoparticles to go through the α phase and the hydrogen-rich β phase (Figure 1c). The use of an aberration-corrected electron microscope has shown the remarkable utility of using light-induced methods to analyze LSPR-related phenomena at sub-2 nm resolution. The work was continued in 2021, when the same group presented a paper discussing the fact that, in plasmonic systems based on Au-PdH_*x*_, energetically unfavorable new nucleation sites are accessible via tailored plasmonic excitation.^39^ In the same year, authors from another research group reported the growth of Au nanoparticles on TiO_2_ substrate under UV–vis irradiation conditions.^23^ The authors observed distinct mechanisms of nanoclusters’ growth—migration and coalescence (shown in Figure 1d) as well as Ostwald ripening. At a similar time, Yu et al. presented photocatalytic self-reduction of CuO and H_2_ generation in an aquatic environment, using their own design of a light-induced liquid cell holder.^40^

A separate and less marked trend is light-induced research, which touches upon the field of life sciences. Ikegami et al.^41^ assessed the effect of the electron beam on cathodoluminescence and the evolution of green fluorescent protein (GFP) under the electron beam conditions. This work is a part of the lateral and somehow controversial trend of observations of light-induced biological interactions using TEM. However, it contributes significantly to the further development and understanding of the damaging effects of beam-induced radiolysis on the investigated processes. The authors added the functionality of a light microscope to TEM along with the possibility of illuminating the sample with a mercury lamp (Figure 2e) to observe the evolution of the photoluminescence and cathodoluminescence spectra of GFP under electron irradiation. This protein is widely used to track the survival of cells or other life processes, and learning about its behavior under the electron beam will allow it to be used in the future for correlative light and electron microscopy (CLEM), also in light-induced processes. In a similar field of life science experiments, I also reported the microstructural effects of methylene blue on microorganisms, which were studied in antimicrobial photodynamic therapy conditions.^42,43^ These works were carried out by placing the optical fiber above the TEM lens,^44^ which facilitated the use of various specimen holders. However, it is difficult to implement it in modern microscopes characterized by a significant accumulation of additional systems and detectors.

It is clear that, despite nearly 20 years of trials, it cannot be said that the described light-induced in situ TEM is an easily available research technique. Customized solutions typically consist of a light fiber illuminator, either built into the sample holder^18,25,29,40,45^ or inside^16,30,37^ or above^44^ the objective pole piece of the TEM. The most popular alternative, based on researchers’ creativity, is the reverse use of cathodoluminescence fixtures that use a parabolic mirror to collect the light generated by the sample.^22,39^ New and still not very popular alternatives are commercially available illuminators^46^ or specimen holders.^23,47^ Most of the possibilities of how to provide light to the specimen are summarized in Figure 3.

**Figure 3:**
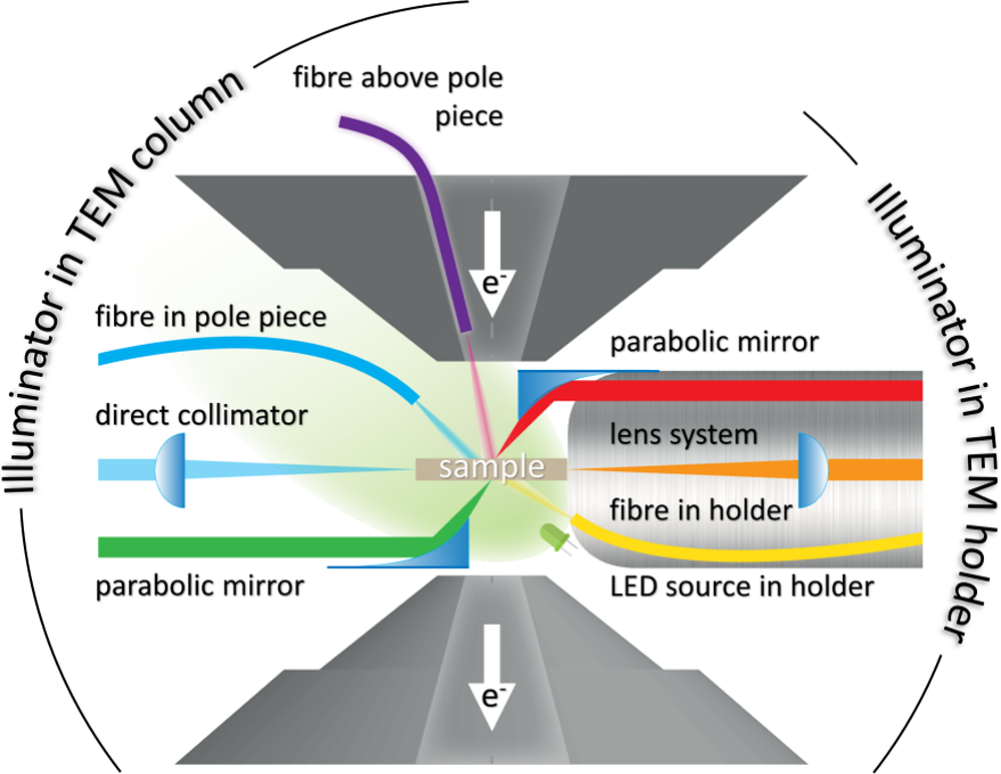
Scheme of different ways to illuminate a sample inside the transmission electron microscope.

There is no universal or perfect solution to light-induced experiments. The TEM sample can receive light from many directions, and each of the methods discussed has its own advantages and disadvantages. A fairly universal solution seems to be delivering the light through the lens system or fiber through the specimen holder. This approach was already combined with micromechanical manipulators to achieve photoelectrical characterization of single ZnO wires^19,33^ or nanoscale heterojunctions.^31,32,38^ On-site fabricated holders seem to be a natural way of popularizing the method, but they have a significant disadvantage. In the context of, for example, catalysis, researchers willingly use not only light but also a liquid or gaseous environment around the sample, or they may even increase its temperature. These types of techniques usually require the use of commercial specimen holders, not equipped with optical fibers. The possibilities of using reactive gas together with heating and lighting were joined in one holder only in the Chip-Nova gas/optics/heating holder.^48^ Optical fibers were also installed in liquid cell holders, both prototype^40^ and commercial.^47^ The obvious advantage of this solution is that there is no need to modify the microscope, which could be expensive and is not always supported by the manufacturer. It is important to distinguish between methods of light supply based on optical fibers and optics. The first solution is definitely easier and provides the freedom to connect various light sources. However, it has significant limitations. Single-mode fibers, suitable for high-frequency pulsed lasers, fit narrow light wavelength ranges when universal multi-mode fibers may not be suitable for carrying high-frequency light. A universal solution for pulsed lasers and a wide range of wavelengths is the use of transparent windows and lenses, which, however, requires much more complex assembly and mechanics. The latter solution seems to be more appropriate in the case of permanent installation in a TEM column. The use of a fiber with a small numerical aperture (NA) provides a narrower beam of light with a greater intensity, but the NA must also be adapted to the parameters of the light source used. Another method is to provide light with parabolic or flat mirrors, placed above^24,49^ or below^41^ the holder or embedded inside it.^45,50^ The parabolic mirror is the standard configuration for cathodoluminescence scanning TEM (STEM) measurements, ensuring maximum collection of light generated by the sample. This guarantees that the illumination is focused on a narrow sample area when the system is used to introduce light. On the other hand, a narrower spot means it is more difficult to align the illumination and observation site. However, the positioning of the mirror below the sample has another significant disadvantage. Although it works well in STEM mode, in conventional TEM this could interfere with the objective aperture, important during continuous imaging. Mirrors also require a relatively large amount of space between the microscope’s pole pieces; nevertheless, this may not be an obstacle to providing atomic resolution images in specific systems.^22,39^

Several users’ own modifications were based on supplying light to the sample with the use of external optical fibers not connected to the holder. They were sometimes brought within^29^ or above^44^ the top objective pole piece. The latter of these cases potentially introduces the most image aberrations due to its proximity to the electron beam and should only be used in low-resolution solutions. However, its advantage is that the sample can be evenly illuminated at an angle close to 90°, similarly to mirror devices. Optical fibers connected from the side may require precise machining^29^ or even tapering^25^ to create the optimal shape of the light beam. The location of the optical fiber inside the pole piece has much less impact on the image. However, in some systems, illumination from the side may result in nonuniform light coverage of relatively large sub-micro- and microparticles. Sometimes it also requires a wedge-like shape of the sample.^25,35^ An interesting solution, although limited only to continuous light, is the use of a LED source within the sample holder.^19,38^ An extension to this solution may be the installation of LEDs on the existing SiN/Si chips used in liquid and biasing holders. This would easily turn the existing holder into a light-induced system. A promising solution for the highest light intensities seems to be the introduction of a light microscopy lens near the sample, which will focus the femtosecond laser beam on the smallest possible diameter of the sample, making it possible to observe multi-photon phenomena.

The basic requirement for introducing light into TEM is to ensure vacuum tightness and precise positioning of the light beam so that it hits the area of electron observation. In the case of self-produced accessories and holders, it is necessary to remember to protect the operator against X-rays and to carry out measurements of ionizing radiation. Determining the real dose of light provided by the system is much more challenging. This requires measurement of the total light energy (provided by a photoelement placed in the plane of the sample) and the diameter of the light spot. In the case of a narrow space of the pole piece, a similar measurement can be made outside the microscope by copying the system geometry. Precise measurement of the amount of light is important because only a small fraction of the energy is delivered to the sample compared to the electron beam. In addition to introducing new object aberrations near the electron beam, the effect of the light beam on the sample temperature should be also considered. A typical vacuum environment thermally isolates the sample, which is used in some systems to intentionally heat the sample while observing^25,36,45^ or cleaning the sample.^51^ Therefore, the high light intensity can lead to inadvertent heating of the sample, leading to distorted conclusions. However, this type of temperature rise can be identified by greater than usual sample drift. The increase in temperature during continuous illumination justifies the use of pulsed lasers, other benefits of which will be discussed in the following paragraphs.

The influence of the sample’s environment on the light-induced experiments should also be determined. The first successful light-induced approach focused on interface reactions between two solid materials, such as the hydrocarbons that covered TiO_2_.^16^ The next step led to ionic liquids, which enabled the exchange of ions, but in an environment far from natural.^17^ Photomechanical studies have been successfully carried out in a vacuum environment;^18,19^ however, typical photocatalytic reactions require an appropriate medium for the transport of electrons and ions. Therefore, ETEM quickly became a natural option for similar experiments, allowing the use of a low-pressure environment of water vapor,^21,26,28,30^ O_2_,^23^ or H_2_.^22,39^ A liquid cell sample can offer faster and more effective observation of photoinduced processes, where, for example, it is possible to observe the formation of gaseous H_2_^37,40^ in an aqueous environment or the microstructural impact of photosensitive substances such as methylene blue.^42,44^ The liquid cell environment, apart from the advantage of the developed interface surface for light-induced changes, has one significant disadvantage. A significant amount of H_2_O can be radiolyzed under the influence of an electron beam, significantly disturbing the reaction environment.^52,53^ This can be minimized by using the medium flow but, in turn, requires the use of holders with SiN windows, which, combined with a significant thickness of the liquid, scatter the electron beam and reduce the available resolution. The graphene liquid cell offers better imaging properties but does not allow for the exchange of the medium. We can also observe changes at the interface of two solid materials in a vacuum, such as TiO_2_ and Au,^23^ but here it should also be remembered that any dose of electrons causes damage such as knock-on displacement, ionization, and radiolysis.^54^

In addition to the phenomena of fluorescence and phosphorescence, the phenomena of light-induced mechanisms may occur, among them switching between the cis and trans forms, electron–hole pair generation, breaking existing chemical bonds, or exposing reactive functional groups. Many of these light-induced effects can also be driven (often more efficiently) by the electron beam during observation, which has already been the subject of extensive analyses.^53−55^ For this reason, some of the first examples of in situ imaging used illumination of the sample for 3 h with an intensity of 10 W/cm^2^,^16,17^ 5 h with an intensity of 6 W/cm^2^,^26^ or even 40 h with an intensity of 1.46 W/cm^2^.^32^ In the discussed cases, the authors imaged the sample at specified intervals, limiting the electron influence only to the observations. Ionizing radiation dose (*D*) is defined as the absorption of one joule of radiation energy (*E*) per kilogram of matter (*m*):
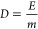
1

Assuming a sample of H_2_O solution with a thickness of 2 μm, 20% sample absorption, and the highest literature continuous light intensity of 10 W/cm^2^,^16,17^ the sample receives a dose of 0.1 Gy every second, i.e., 360 Gy/h. To compare this value with the effect of the electron interaction during imaging, we could use the method of converting the surface electron dose [e^–^/A^2^] described in ref (55):
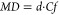
2where *MD* is the ionizing radiation dose [MGy], *d* is the electron fluence [e^–^/A^2^], and *Cf* is the correction factor. For H_2_O, *Cf* = 4.36 at an accelerating voltage of 100 kV and *Cf* = 6.59 at an accelerating voltage of 200 kV. For the dose rate of 200 e^–^/A^2^·s used for medium magnification imaging in conventional TEM, every second the H_2_O sample receives an ionizing radiation dose equal to 6.59 × 10^10^ Gy at 200 kV. This is more than 8 orders of magnitude higher than the exemplary hourly light illumination. The matter looks slightly better when the size of the absorbed energy quanta is taken into account. Although the electron accelerated by a voltage of 200 kV has an energy of 2 × 10^5^ eV, its dominant loss of elastic scattering is less than 0.1 eV, and for inelastic scattering, it could reach tens of eV, representing the plasmon resonance of the valence electrons.^56^ A single photon with the wavelength of 350 nm carries the energy of 3.542 eV, but it can be completely absorbed only by the molecule. The small amount of absorbed photons, which for the illumination parameters described above is 3.53 × 10^–6^ [1/A^2^·s], requires long exposure times, significantly affecting the temperature of the sample. On the other hand, the situation could be much more favorable when a pulsed laser is used. Considering the illumination used in refs (22 and 39), the 3.8 mW mean beam power on a 200 μm diameter spot gives the mean intensity of 121 W/cm^2^. The peak pulse power density (*P*_peak_) could be estimated from the following equation:
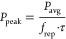
3where *P*_avg_ is average beam power, *f*_rep_ is repetition rate, and τ is pulse length. For a repetition of 78 MHz and 120 ps pulse length, *P*_peak_ reaches 1.3 × 10^9^ W/cm^2^. By using the wavelength appropriate to the LSPR and the fact that the excitation time exceeds the pulse period, the authors significantly multiplied the probability of observing light-driven processes. This type of illumination could help to avoid unnecessary heating of the material, typical of long exposure to the continuous laser and good thermal insulation of the sample surrounded by vacuum or low-pressure reactive gas. It is worth noting that, in order to ensure high-resolution observation, the authors of refs (22 and 39) cared about maintaining a low dose of electrons, reaching 10 e^–^/A^2^ at relatively high magnifications. This also tilts the probability toward light-induced phenomena; however, it cannot be ignored while analyzing the results. Lu et al. used an even lower electron dose of 3 e^–^/A^2^ per frame and blanked the beam for a few hours between exposures, ensuring that the electron beam impact is minimized during comparatively weak excitation by a continuous light source. The interaction of electrons with matter is a complex topic that has been described in detail elsewhere,^52−54,56,57^ and this type of reading should be a must for anyone considering light-induced experiments.

Systems suspended in liquids are potentially attractive for future observations of photocatalytic and plasmonic reactions. However, it should be remembered that the main part of sample damage in the interaction of electrons with common solvents is caused by radiolysis, the appearance of free radicals, and the accompanying change in the composition of the solution.^52^ Therefore, there is no doubt that the number of electrons should be minimized to reduce the probability of excitation of the material. The essential techniques include the low-dose method, in which electrons hit the sample only during imaging, usage of liquid flow, the introduction of sensitive electron detectors, and the use of the pulse lasers, as justified above. The value of a large number of comparative experiments conducted under conditions of varying intensity of the light and electron beams cannot be underestimated. It is particularly important to introduce control experiments when the interaction of light inside the electron microscope is conducted over a longer time with a blanked electron beam.

Phase contrast is a promising candidate to allow high-resolution imaging using a lower than typical electron dose, achievable with phase plates.^54,56^ These devices are now used in cryogenic transmission electron microscopy (cryoEM), enabling similar resolution of 3D protein imaging using only a fraction of the data and acquisition time. Phase plate usage seems to be particularly promising for the imaging of organic matter in liquids, which is characterized by electron scattering similar to that of the surrounding solvent. Using this exemplary method of contrast improvement, in addition to the most sensitive detectors, will allow the use of a minimal dose of electrons, clearly separating the effects induced by light from the effects induced by the electron beam.

Up to now, light-induced in situ TEM has been used occasionally, so it is not necessary to indicate very distant prospects for its use. The first choice for future research could be interesting photocatalytic systems, including the most studied TiO_2_^16,17,23,28,30,37,38^ and ZnO.^13,19,20,24,26,27,32,33^ On the subject of photocatalysis, experiments in reactive gases in ETEM and liquid cell TEM are already widespread.^58^ Many of the processes described in the literature do not need to be just electron-beam-driven but can be light-induced as well. Heterogeneous catalysis processes seem to be extremely promising because in their case the gaseous or solid products of catalysis could be easily observed in liquid media (liquid cell TEM) or the solid products of catalysis could be observed in the gaseous environment (ETEM).^59^ A relatively poorly understood area is the observation of LSPR effects.^22,39^ The wider use of pulsed lasers may lead to the observation of not only single-photon but also multi-photon processes in the future. In this case, one should expect an increase not only in classical TEM but also in ultrafast TEM^11,12,60^ methods. Material processing methods can benefit from the development of photothermal in situ experiments of laser ablation, sintering, and welding.^36^ We cannot omit the growing importance of research on the essential nature of the quantum interaction of light and electrons.^61−63^ Such interaction was also manifested in the creation of effective laser phase plates, promising in the field of cryoEM systems development.^64^ There are also promising possibilities for magnetic Lorentz microscopy,^65^ holographic imaging of photosensitive semiconductors,^66^ and further development of low-dose electron imaging methods, leading to a situation in which we will be able to take several electron images of the microorganism in situ before reaching lethal conditions.^67^ In such a case, it is expected that the described techniques will be used more widely in the field of life sciences, for example in the field of antibacterial photodynamic therapy and in research on photosynthesis.
